# Pancreaticobiliary maljunction diagnosed long after laparotomy in the neonatal period for annular pancreas: report of a case

**DOI:** 10.1186/s40792-019-0572-2

**Published:** 2019-02-01

**Authors:** Naoya Sato, Tatsuo Shimura, Akira Kenjo, Takashi Kimura, Junichiro Watanabe, Makoto Muto, Shigeru Marubashi

**Affiliations:** Department of Hepato-Biliary-Pancreatic and Transplant Surgery, Fukushima Medical University, Hikarigaoka-1, Fukushima-shi, Fukushima, 960-1295 Japan

**Keywords:** Annular pancreas, Pancreaticobiliary maljunction, Chronic pancreatitis

## Abstract

**Background:**

Although annular pancreas concurrent with pancreaticobiliary maljunction has rarely been reported, some reports have pointed out a possibility that both anomalies have a common pathogenesis in pancreatic development. We herein report a case with pancreaticobiliary maljunction diagnosed long after surgical treatment for annular pancreas.

**Case presentation:**

A 34-year-old female, with a surgical history of duodenal obstruction due to annular pancreas in the neonatal period, was referred to our hospital for further examination of chronic pancreatitis. Endoscopic retrograde cholangiopancreatography and magnetic resonance cholangiopancreatography revealed choledocholithiasis, pancreatic lithiasis, and pancreaticobiliary maljunction without biliary dilatation. Choledocholithotomy and cholecystectomy were performed, and highly elevated levels of amylase in bile from the common bile duct were found intraoperatively.

**Conclusion:**

The present case highlights a possible association of pancreaticobiliary maljunction in a patient with annular pancreas.

## Background

Pancreatic development, involving the generation of the liver buds, occurs at approximately 4 weeks of gestation. Subsequently, the hepatic diverticulum separates into two protruding lobes, the left and right lobes. After the left lobe quickly retracts, the right lobe becomes the ventral pancreatic anlage. At approximately 6–7 weeks of gestation, in association with the 90-degree rotation of the stomach and duodenal loop, the ventral anlage moves to the rear with rotation around the duodenum and then ventrally approaches the dorsal anlage. Finally, both the ventral and dorsal anlages assimilate to form the pancreas [1]. Thus, pancreatic development is so complicated that congenital abnormalities often occur.

Annular pancreas and pancreaticobiliary maljunction are well-known abnormalities. In annular pancreas, the descending part of the duodenum is surrounded by a ring of pancreatic tissue continuous with the pancreas head. In pancreaticobiliary maljunction, anatomically, the pancreatic and bile ducts join outside the duodenal wall [2]. The causes of both diseases remain unclear; however, a previous study pointed out the possibility that the abnormality of the ventral pancreatic anlage during the pancreatic development might be a common pathogenesis [3]. In the present report, we describe a case of pancreaticobiliary maljunction diagnosed 30 years after surgical treatment for annular pancreas with a review of literature.

## Case presentation

A 34-year-old woman presented to a physician with chief complaints of abdominal pain and fever. The physician suspected acute exacerbation of chronic pancreatitis and referred the patient to the Department of Gastroenterology, Fukushima Medical University Hospital. The patient had a medical history of duodenal atresia treated by emergent surgery 2 days after birth, and annular pancreas and malrotation of the intestine, for which duodenoduodenal anastomosis with Ladd’s procedure was performed. Operative findings revealed no dilatation of the common bile duct. At 9, 23, and 25 years of age, she suffered from repetitive acute pancreatitis and required hospital treatments. Since she had recurrent epigastralgia and back pain, she was diagnosed as having chronic pancreatitis and was prescribed with oral drugs. She was a non-smoker and reported occasional alcohol consumption with no relevant family history.

**Fig. 1:**
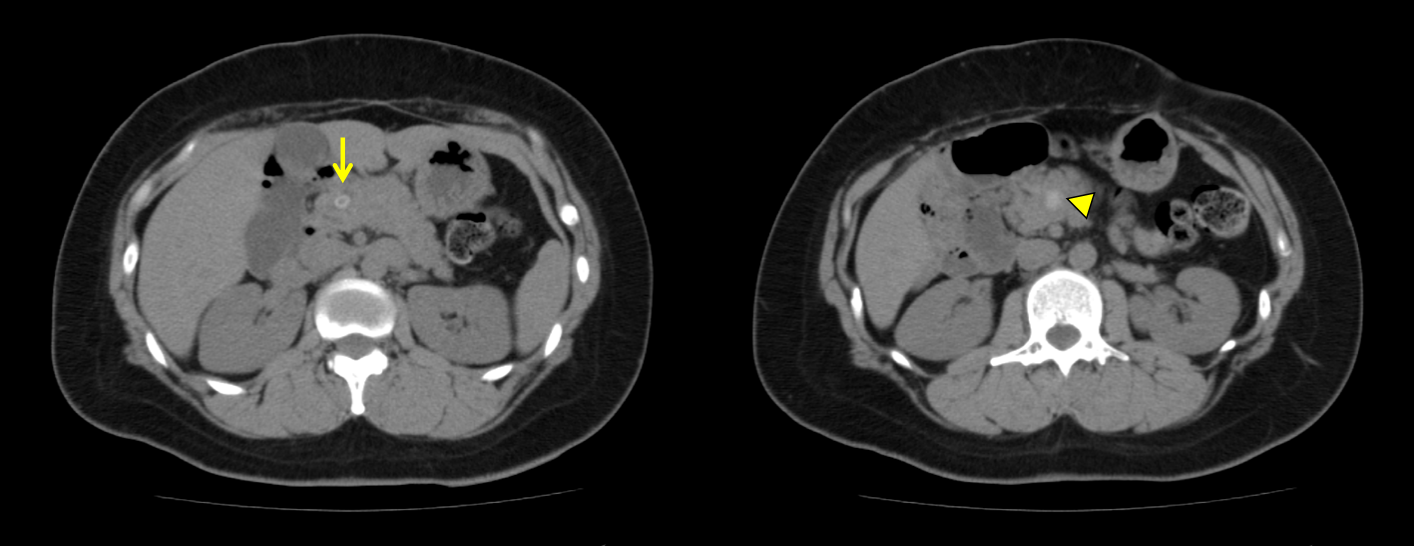
Findings of computed tomography. The choledocholithiasis (arrow) and pancreatic calculi (triangle) was detected

**Fig. 2:**
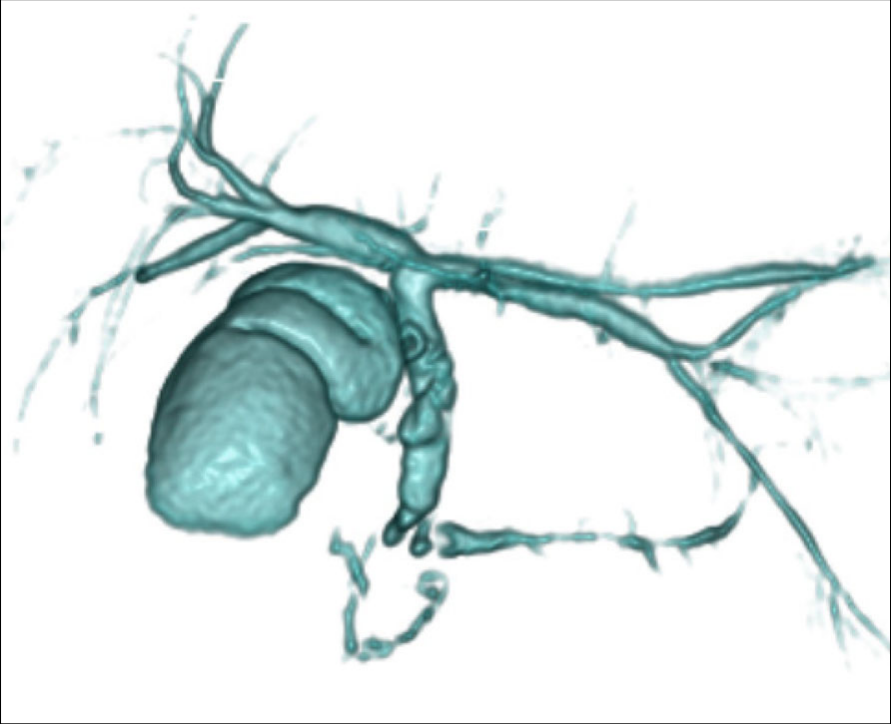
Findings of magnetic resonance cholangiopancreatography. The annular pancreas duct around the second portion of the duodenum and filling defect of the main pancreatic duct in the pancreas head, shown by magnetic resonance cholangiopancreatography

**Fig. 3:**
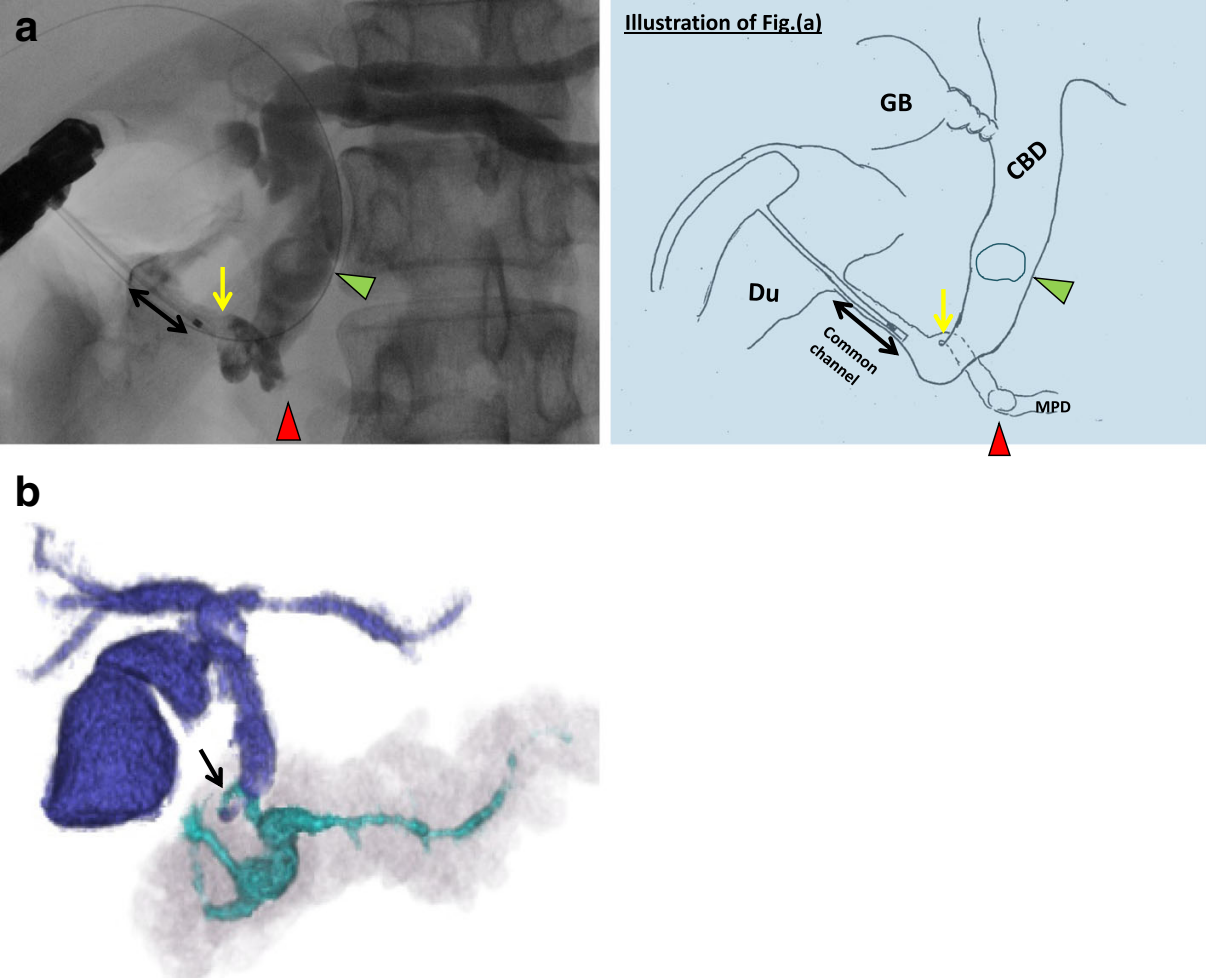
Findings of endoscopic retrograde cholangiopancreatography and three-dimensional cholangiopancreatography by computed tomography data. A filling defect in the common bile duct due to choledocholithiasis (green triangle) and the main pancreatic duct in the pancreas head (red triangle), detected by endoscopic retrograde cholangiopancreatography; ERCP (**a**). And, the pancreatic duct joined near the lower common bile duct (arrow), and the common channel (two-way arrow) was revealed. Three-dimensional cholangiopancreatography (**b**) revealed joint portion of pancreatic duct and common bile duct and annular pancreatic duct. Pancreas duct distal to the filling defect was slightly dilated

Laboratory data from blood samples taken at the patient’s first visit to our department exhibited slight elevation of hepatic and biliary tract enzymes (glutamic oxaloacetic transaminase 53 IU/L, glutamic pyruvic transaminase 94 IU/L, alkaline phosphatase 446 IU/L, gamma-glutamyl transpeptidase 259 IU/L). Abdominal computed tomography (CT) showed a small round stone, approximately 9.3 mm in diameter, in the common bile duct, and a pancreatic calculus, approximately 14 mm in diameter, in the pancreatic head duct (Fig. 1), causing slight dilatation of the distal pancreatic duct. Abdominal ultrasonography showed no dilatation of the intrahepatic bile duct and no thickness of the gallbladder wall. Magnetic resonance cholangiopancreatography (MRCP) revealed annular pancreas around the second portion of the duodenum (Fig. 2). Endoscopic retrograde cholangiography (ERCP) was performed to determine the cause of the pancreatitis. ERCP showed a round filling defect caused by the above-mentioned stone and pancreatic calculus (Fig. 3a). The distal portion of the common bile duct was bended due to the previous surgical procedures for annular pancreas in the neonatal period. Pancreaticobiliary maljunction was suspected because the contrast medium refluxed to the pancreatic duct during cholangiography (Fig. 3a). The common channel could not be measured exactly, because of the bended distal portion of the common bile duct. The amylase level in bile juice obtained during ERCP was elevated to 2513 U/L. The 3D-cholangiography reconstructed from the CT data (Fig. 3b) revealed that the pancreatic duct joined the common bile duct at the bend. Preoperative endoscopic ultrasonography was not performed in the present case. As endoscopic sphincterotomy was difficult to perform because of the deformed duodenum due to the previous surgery, only a balloon dilatation of Vater’s papilla was performed. Furthermore, removal of the common bile duct stone could not succeed. Then, we decided to perform surgical treatment. Although intraoperative cholangiography via the cystic duct showed a filling defect in the common bile duct, cholangioscopy via a small incision on the common bile duct showed no stones. Thus, we concluded that that the stone had passed spontaneously. On the basis of the preoperative findings, high levels of amylase in the gallbladder (22,300 U/L), and the presence of a relatively long common channel detected by cholangiography, our patient was diagnosed as having a pancreaticobiliary maljunction without bile duct dilatation. We performed a cholecystectomy in the laparotomy and placed a C-tube in the common bile duct. In histopathological findings, reactive lymph follicle and lymphocytic infiltration were observed around Rokitansky-Aschoff sinus in the gallbladder with no neoplastic lesion in the mucosa.

The postoperative course was uneventful. The bile juice drained from the C-tube showed elevated levels of amylase (32,572 U/L). On postoperative day 13, the C-tube was removed, and the patient was discharged on postoperative day 17. At time of writing, 17 months after surgery, there has been no relapse of pancreatitis. However, medical treatment has been maintained for chronic pancreatitis in this patient.

## Discussion

The frequency of annular pancreas is reported to be approximately one in 1000 patients who underwent ERCPs [4] and less than 1.5 in 10,000 births [5]. Annular pancreas is known to occur with congenital deformities, including Down’s syndrome (18.8%), malrotation of the intestine (18.2%), duodenal atresia (17.5%), and heart malformation (17.5%) [6]. In regard to pancreaticobiliary maljunction, a patient with combined annular pancreas and pancreaticobiliary maljunction is extremely rare. There have been only 12 reported cases of patients with comorbid condition of annular pancreas and pancreaticobiliary maljunction [3, 5–13]. Characteristics of these 12 cases are summarized in Table 1. The male-to-female incidence ratio was 0.5. There were two cases concomitant with malignant biliary tract tumors: both were adults, one case with dilated biliary tract and bile duct cancer [9] and the other with gallbladder cancer without bile duct dilatation. Considering that these two cases have dilated common bile duct (CBD), it can be speculated that presence of dilated CBD might be a risk factor for carcinogenesis. Biliary tract cancers develop about 15–20 years earlier in patients with pancreaticobiliary maljunction than in individuals without pancreaticobiliary maljunction [14]. Carcinogenesis is strongly associated with stasis of bile intermingled with refluxed pancreatic juice, by which epithelial cell in the biliary tract are damaged, developing hyperplastic change with increased cell proliferation. Therefore, it is necessary to consider a potential comorbidity of pancreaticobiliary maljunction at the time of diagnosis of annular pancreas, especially in pediatric patients. Not a few pediatric patients with annular pancreas may be observed without examination of the biliary tract. In pediatric patients with symptoms suspected of chronic pancreatitis or gallstones after surgery for annular pancreas, detailed examination of biliary tract for investigating pancreaticobiliary maljunction should be performed. In recent years, the efficacy of MRCP as a noninvasive modality for detection of a pancreaticobiliary maljunction and annular pancreas has been reported [15]. In this regard, however, Japanese clinical practice guidelines for pancreaticobiliary maljunction define the diagnostic criteria as having a long common channel detected by cholangiography and a junction of the pancreatic duct and bile duct outside the duodenal muscular wall. Elevated amylase levels in the bile duct and gallbladder are only used as supporting findings for the diagnosis [16]. The diagnostic problem of our case was that it was impossible to measure the length of the common channel exactly.

**Table 1:** Summary of patients of annular pancreas concurrent with pancreaticobiliary maljunction reported in Japan

No.	Author	Year	Age (years old)	Sex	CC on set	Dilation of CBD	Cancer	Surgical treatment
1	Shimizu [7]	1988	25	Female	Abdominal pain	Cystic	None	Resection of the BD
2	Komura [8]	1992	2	Female	Abdominal pain	None	None	Duodenoduodenostomy resection of the BD
3	Okada [6]	1993	12	Male	Abdominal pain	Fusiform	None	Duodenoduodenostomyresection of the BD
4	Okada [6]	1993	3	Female	Abdominal pain	Fusiform	None	Duodenoduodenostomy resection of the BD
5	Mukuta [9]	1993	46	Female	Jaundice	Cystic	BD cancer	PD
6	Matsuyama [10]	1993	26	Female	Abdominal pain	Fusiform	None	Resection of the BD
7	Ochiai [11]	1997	65	Female	Abdominal pain	None	GB cancer	Resection of the BD cholecystectomy
8	Sugimoto [12]	2002	2	Male	Hepatic dysfunction	Fusiform	None	Duodenoduodenostomy resection of the BD
9	Owari [5]	2004	8	Female	None	Fusiform	None	Duodenoduodenostomy resection of the BD
10	Nomura [13]	2007	61	Male	Nausea	None	None	Duodenoduodenostomy cholecystectomy
11	Kodama [3]	2011	80	Male	Pancreatitis	Fusiform	None	None
12	Our case	2018	34	Female	Pancreatitis	None	None	Duodenoduodenostomy cholecystectomy

*Abbreviations*: *CC* chief complaint, *CBD* common bile duct, *BD* bile duct, *PD* pancreaticoduodenectomy

As mentioned in the “Background” section, pancreatic development is so complicated that congenital abnormalities often occur. The developmental mechanism of annular pancreas and pancreaticobiliary maljunction has been reported to have a similar malformation in the early phase of pancreatic development [3], especially in *ventral pancreatic anlage*. As for the cause of annular pancreas, Baldwin’s theory explains typical causes of this condition, in which the left lobe of the ventral pancreatic anlage (which normally disappears) remains and grows as forming a ring [17]. Kamisawa [1] supported Baldwin’s theory because many cases of annular pancreas exhibited thin pancreatic parenchyma with histologically proven fibrotic tissues, indicating that annular pancreas is formed from the left lobe of ventral pancreatic anlage (which normally regresses). As for the cause of pancreaticobiliary maljunction, Oi claimed that the pathological mechanism of this disorder should be an abnormal junction of the bile and pancreatic ducts in ventral pancreatic anlage, because a dorsal pancreatic duct was almost always normal [18]. These concepts can support a supposition that a combination of annular pancreas and pancreaticobiliary maljunction could occur with some frequency.

The clinical presentation of annular pancreas is classified into two types: infant/pediatric-type and adult-type. Most patients with the infant/pediatric-type can be diagnosed soon after birth because its onset starts with frequent vomiting due to duodenal stenosis or obstruction by annular pancreas. Thus, duodenal bypass surgery must be performed within approximately 2 days of birth [19]. On the other hand, since the pathological condition of adult-type annular pancreas is chronic inflammation of annular pancreas, this type has only minor or nonspecific symptoms, such as feelings of epigastric fullness, uneasiness, nausea, and vomiting. However, some adult-type cases have been reported to present obvious symptoms due to duodenal stenosis, as well as subsequent peptic ulcers, pancreatitis, gallstones, and pancreatic calculus caused by an abnormal pancreatic conduit [10, 13].

In the present case, acute exacerbation of chronic pancreatitis has not been relapsed after a balloon dilatation of Vater’s papilla and cholecystectomy. The reason that acute exacerbation of chronic pancreatitis has not been relapsed with existing pancreatic calculi is unclear. It can be speculated that CBD stone was associated with repeated acute exacerbation of chronic pancreatitis. In this case, the main pancreatic duct is slightly dilated due to pancreatic calculus 17 months after surgery. Therefore, long-term follow-up for chronic pancreatitis with medical treatment must be required.

## Conclusion

In the present case, we experienced a patient diagnosed with pancreaticobiliary maljunction 30 years after annular pancreas surgery. Both annular pancreas and pancreaticobiliary maljunction are considered to be congenital abnormalities occurring during development of the pancreas. Because pancreaticobiliary maljunction has potential for developing biliary tract cancer, patients with annular pancreas must be followed up with considering the possibility of coexisting pancreaticobiliary maljunction.

## Abbreviations

CBD: Common bile duct
CT: Computed tomography
ERCP: Endoscopic retrograde cholangiopanceatography
MRCP: Magnetic resonance cholangiopancreatography
